# Single-cell RNA sequencing provides novel insights to pathologic pathways in abdominal aortic aneurysm

**DOI:** 10.3389/fcvm.2023.1172080

**Published:** 2023-05-23

**Authors:** Jack Bontekoe, Bo Liu

**Affiliations:** ^1^ Division of Vascular Surgery, Department of Surgery, University of Wisconsin-Madison School of Medicine and Public Health, Madison, WI, United States; ^2^ Department of Cellular and Regenerative Biology, University of Wisconsin-Madison School of Medicine and Public Health, Madison, WI, United States

**Keywords:** abdominal aortic aneursym, single-cell RNA sequencing, transcriptomic, inflammation, vascular smooth muscle cell (VSMC)

## Abstract

There is gaining popularity in the use of single-cell technology and analysis in studying the pathogenesis of abdominal aortic aneurysm (AAA). As there are no current pharmacologic therapies for impeding aneurysm growth or preventing AAA rupture, identifying key pathways involved in AAA formation is critical for the development of future therapies. Single-cell RNA sequencing (scRNA-seq) technology provides an unbiased and global view of transcriptomic characteristics within each of the major cell types in aneurysmal tissues. In this brief review, we examine the current literature utilizing scRNA-seq for the analysis of AAA and discuss trends and future utility of this technology.

## Introduction

Abdominal aortic aneurysm (AAA) is a complex multifactorial pathologic dilation of the abdominal aorta. AAAs are prevalent in roughly 1–2% of men over the age of 65 years, and nearly 0.5% of women over 70 years old, however, AAA rupture is the leading cause of death in the United States (1, 2). Aneurysms progressively dilate over time, and once reaching a threshold size, elective surgical repair with either open surgery or endovascular graft placement is recommended. Due to the lack of effective pharmacological therapies, surgical intervention remains the only available treatment for this disease. Without intervention, continued AAA sac expansion increases the risk of aneurysm rupture, which carries significant morbidity and mortality.

Due to the complex interplay of biochemical, hemodynamic, genetic, and environmental mechanisms, investigating the pathophysiology of AAA presents a challenge. Arterial wall damage related to advanced age, smoking, hypertension, male gender, and family predisposition results in an accumulation of chronic inflammation, oxidative stress and faulty repair mechanisms (2, 3). Irreversible changes in the vessel wall due to smooth muscle cell death, the activation of matrix metalloproteases, and altered extracellular matrix composition cause progressive dilation and weakening of the aortic wall (2). Disrupted arterial flow within the aneurysm sac places additional biomechanical stressors on the wall, further contributing to the pathologic process (4). Despite the identification of many mechanisms involved in AAA, there is limited understanding in the way these processes are interrelated and regulated at the cellular and molecular levels.

Single-cell RNA sequencing (scRNA-seq) is a technique used to analyze gene expression at the level of individual cells. Compared to conventional RNA sequencing, which analyzes an average gene expression in bulk tissue samples, scRNA-seq provides a more detailed and precise evaluation of cell heterogeneity within samples, as it can identify rare cell types and even predict cell-cell signaling pathways. Since the discovery of single-cell RNA sequencing (scRNA-seq) by Tang et al. in 2009, the use of this technology has become increasingly embraced in recent years by pathophysiological studies (5). Analyses of RNA transcripts have further demonstrated the vast complexity and heterogeneity of cells involved in cardiovascular disease. However, its use in the evaluation of AAA was unknown only until recently in 2018 (6). Today, scRNA-seq in AAA is gaining popularity, partially through the availability of public datasets. In this study, we review the current literature on scRNA-seq in AAA to demonstrate the potential of this technology in studying aneurysmal disease, and its challenges.

## Methods

A literature search of publications regarding scRNA sequencing on AAA was performed using the PubMed search engine MEDLINE database. Search terms included (“single-cell RNA” OR “scRNA-seq”) AND “abdominal aortic aneurysm(s)” queried within all fields of text in order to capture the largest number of articles. Only original studies with full text published prior to 2023 were included. Studies were excluded if no new primary scRNA-seq data or in-depth analysis of existing datasets were performed. The model or tissue specimen to which scRNA-seq was performed, the number of cells and clusters, and the major scRNA-seq specific findings were then compared.

## Results

The above search terms yielded 17 results, of which 2 studies were excluded. One study was excluded as the authors used a previously published scRNA-seq database to identify the presence of an RNA sequence of interest instead of analyzing the dataset (7). A review article was also excluded as it did not contain primary data (8). Of the remaining 15 manuscripts, 9 studies collected primary scRNA-seq data (Table 1), and 6 studies reanalyzed available scRNA-seq databases (Table 2).

**Table 1:** Studies involving primary collection and analysis of single-cell RNA sequencing of AAA tissue.

Author	Year	Tissue source/model	Cells/clusters	Major findings by scRNA-seq
Hadi et al. (6)	2018	Murine—*Ntn1-*deficient vs. WT chimeric *ApoE^−/−^*mice at day 28 of continuous subcutaneous infusion of Ang II (suprarenal aortic tissue)	• Immune cell clusters: 5	• 5 subpopulations of immune cells were identified by *t*-distributed stochastic neighbor embedding (*t*-SNE), to which Mø expressed the highest percentage of necrotin-1 (*Ntn1*) positivity • Aneurysm Mø enriched with *Ntn1* expressed high pro-inflammatory and angiogenic markers compared to Mø with low *Ntn1* expression • Pathway analysis using DAVID software predicted crosstalk between netrin-1 and calcium signaling regulation, potentially linking macrophage secretion of netrin-1 to VSCM calcium flux and sequential production and activation of MMP3 within AAA
Yang et al. (9)	2021	Murine—C57BL/6J mice at day 4 post-sham or perivascular application of CaCl_2_ (infrarenal aortic tissue)	• Cells: 3,896 • Sham: 2,537 • CaCl_2_: 1,359 • Clusters: 12	• AAA aortas had reduced SMC cell density, increased macrophages, and reduced percentage of fibroblasts. The largest cell population within AAA tissues were fibroblasts • *Cebpb* (encoding for basic leucine zipper transcription factor CEBP-B) was the most upregulated gene in AAA; *Spp1* (encoding osteopontin) showed largest fold increase compared to sham • Macrophages were composed of 3 distinct populations; Mø-1 demonstrated an anti-inflammatory gene profile, Mø-2 shown a classic inflammatory profile, while Mø-3 had enriched proliferation pathways
Zhao et al. (10)	2021	Murine—C57BL/6J mice at days 7 and 14 post-sham or peri-adventitial elastase-induced AAA (infrarenal aortic tissue)	• Cells: 4,642 • Sham: 1,509 • 7d: 1,737 • 14d: 1,396 • Clusters: 17	• AAA progression decreased SMC contractile markers, and upregulated pro-inflammatory genes • Immune cell expansion, particularly activated resident macrophages, blood-derived monocytes, inflammatory macrophages, and reparative macrophages
Davis et al. (11)	2021	Human—infrarenal AAA (*n *= 4) vs. controls (aortic tissue isolated from patients with non-aneurysmal atherosclerotic disease undergoing open aortobifemoral bypass; *n *= 2)	• Clusters: 21	• Expression of JMJD3, an epigenetic histone demethylase, is higher in human AAA tissues than controls • Pathway analysis was performed on DEGs from monocyte/macrophage populations. AAA monocytes shown greater upregulation of inflammatory genes involving cytokine-mediated signaling, NF-kB transcription activity, antigen processing, and T lymphocyte co-stimulation, as well as a reduction in anti-inflammatory genes • Screening of altered epigenetic enzymes in AAA monocyte/Mø showed an elevated expression of *JMJD3*, which was not seen in T or B lymphocytes, similar to findings in murine AAA modeling • Inflammatory pathways and cytokines were elevated in JMJD3^+^ monocytes, compared to JMJD3^−^ monocytes
Li B. et al. (12)	2021	Murine—*ApoE*^−/−^ mice at day 28 of continuous subcutaneous infusion of Ang II vs. sham (suprarenal aortic tissue; *n *= 4–5 per group)	• Cells: • Controls: 7,914 • AngII: 9,338 • Clusters: 15	• AAA tissue shows macrophage heterogeneity among 3 subtypes, with an increase in *Trem2*^+^ osteoclast-like Mø • Gene expression patterns and functions of AAA fibrocytes are distinct from Mø • Co-expression of *Ptprc* and *Col1a2* is distinct to fibrocytes, separate from macrophages, fibroblasts, and SMCs; co-expression levels also identified fibrocytes in a scRNA-seq database of human ascending thoracic aortic aneurysm tissues • GFP-labeled fibrocytes were recruited to AAA tissues and attenuated AAA formation, growth, and elastin degradation
Qian et al. (13)	2022	Murine—*ApoE*^−/−^ mice at day 28 of continuous subcutaneous infusion of Ang II vs. sham (suprarenal aortic tissue; *n *= 4–5 per group)	• Clusters: 9	Enrichment analysis of VSMCs signaling pathways showed upregulation of multiple networks involving actin cytoskeleton regulators, and elevated expression of *Actn2* in AAA VSMCs Expression of *Piezo1,* a mechanosensory ion channel, is concentrated in VSMCs from Ang II-AAA mice MMP3 is enriched in AAA VSMCs, compared to sham
Gao et al. (14)	2022	Murine—healthy male C57BL/6J mice	• Cells: 10,138 • VSMC-like clusters: 11	• Evaluation of normal aortic tissue demonstrated 11 VSMC-like clusters, including 9 VSMC subpopulations, one EC-like VSMC cluster, and one fibroblast-like cluster • Gene ontology (GO) analysis of DEGs showed functional diversity of the 9 VSMC subpopulations; pathway enrichment analysis revealed functional heterogeneity of VSMCs • RGS5, a regulator of G protein signaling molecules, was specifically elevated in the homeostasis associated VSMC subpopulation, which immunohistochemistry revealed RGS5 and Fn positive VSMCs were nearly absent in AAA tissue
Weng et al. (15)	2022	Murine—*ApoE*^−/−^ mice at day 28 of continuous subcutaneous infusion of Ang II vs. sham (suprarenal aortic tissue)	• Cells: >27,000 • Sham: 13,779 • AAA: 14,086 • Clusters: 25	• AAA wall tissue was largely composed of fibroblasts and Mo/Mø by proportion, compared to controls consisting of fibroblasts and endothelial cells. AAA also showed diminished levels of T and B cells • AAA SMCs upregulated secretory marker genes, while contractile markers remained unchanged • AAA induces large heterogeneity of fibroblasts, shown by 10 clusters; expression of *Acta2*, *Taglin*, and *Spp1* suggests differentiation into myofibroblasts • Trajectory analysis of AAA fibroblasts revealed genes with upregulated expression involved in regulation of complement cascade, negative regulation of immune system, and antigen processing and presentation

Ang II, angiotensin II; EC, endothelial cell; Mø, macrophages; MMP, matrix metalloprotease; Mo, monocytes; SMC, smooth muscle; VSMC, vascular smooth muscle cell.

**Table 2:** Studies analyzing previously published AAA scRNA-Seq data and databases.

Author	Year	Model/tissue	Cells/clusters	Major findings by scRNA-seq
Liu et al. (16)	2020	Re-analysis of Ang II-induced murine AAA scRNA-seq database compared to a human AAA mRNA microarray dataset	• Clusters: 5	• Analysis of human AAA mRNA microarray dataset identified DEGs, which were then used to construct a PPI network revealing 3 genes previously reported to be associated with AAA, and 3 novel genes. The Ang II murine AAA scRNA-seq database was analyzed for these results, revealing 4 AAA-linked genes in common—*CANX*, *CD44*, *STAT1*, and *DAXX*
Yang et al. (17)	2022	Comparison of CaCl_2_-, elastase-, or Ang II-induced murine AAA and human AAA scRNA-seq datasets evaluating cell-cell communication	CaCl_2_ • Cells: 3896 • Sham: 2,537 • AAA: 1,359 • Clusters: 12 Elastase • Clusters: 16 Ang II • Clusters: 9 Human • Clusters: 14	• Intercellular communication networks were evaluated in murine and human AAA scRNA-seq databases using CellChat analysis • CaCl_2_ database analysis showed 8,799 ligand-receptor interactions in the sham group, compared to 8,601 in AAA. Outgoing and ingoing signals were highest in the SMC and Fib-1 cell populations. AAA induction increased signaling from Maph-2 to SMC-1 and from DC to SMC-2. Seven signaling pathways were unique to sham, while three pathways were unique to AAA. • Elastase database analysis showed 7,233 interactions in the control group, 10,453 in the Day 7 post-surgery group, and 9,343 in the Day 14 group. AAA increased the communication probability compared to controls, with SMCs as the major signal source and fibroblasts the target. Five signaling pathways were exclusive to controls, while 21 pathways were only expressed in the elastase treatment groups • Ang II database analysis showed fibroblasts and SMC populations were the major signal source while SMC-1 was the major receiver • Human AAA database analysis showed 52 total interactions in the control group, compared to 972 interactions in AAA. Interaction strength was nearly undetectable in the control group. In the AAA group, the SMC and fibroblast populations were the major signal senders, while the NK cell population was the major receiver. Most signaling pathways were expressed in AAA • Eight signaling pathways were altered in all murine models and human AAA. MIF signaling was upregulated in all AAA groups.
Li Y. et al. (18)	2022	Analysis of human AAA scRNA-seq database, GSE166676, compared to transcriptomic data of hypertension and intracranial aneurysm datasets	• Cells: 9,796 • Clusters: 21	• Reanalysis of human AAA database produced 21 cell clusters. The Mo/Mø cluster of 2,102 cells was used for differential analysis between controls and AAA tissues. DESeq2 and FindMarker of Mo/Mø identified 869 DEGs, which were then were utilized to further analyze bulk RNA-seq data of hypertension and intracranial aneurysm datasets
Xiong et al. (19)	2022	Comparison of human AAA scRNA-seq database, GSE166676, to 4 human AAA RNA chip datasets	• Clusters: 20	• DEGs identified in the scRNA-seq dataset were aggregated with DEGs identified by weighted co-expression network analysis (WGCNA) of the RNA chip datasets • *G0S2* was identified as a diagnostic biomarker for early AAA and *HPSE* correlated with rupture risk in large AAAs • Immune infiltration analysis of AAA and normal samples using the CIBERSORT algorithm showed T follicular helper (Tfh) cells were overexpressed in AAA, especially in larger sized aneurysms
Davis et al. (20)	2022	Reanalysis of scRNA-seq data from human infrarenal AAA and control tissues	• Cells: 9,290 • Clusters: 17	• 8 major cell types in were identified in infrarenal AAA: endothelial cells, SMCs, fibroblasts, myeloid cells, T cells, NK, B cells, and plasma cells • The SMC cluster showed high expression of contractile proteins encoded by *TAGLN*, *ACTA2*, and *MYL9*; 3 fibroblast clusters were identified, 2 displaying inflammation activation while one demonstrated high extracellular matrix organization • The majority of the immune cell population was comprised of T lymphocytes • 5 of 8 AAA cell types showed >300 DEGs; SMCs showed the greatest number of DEGs, with upregulated genes involving cell migration/proliferation and TGFß receptor signaling • Comparison of DEGs with GWAS results from AAA patient peripheral blood showed the aneurysm-associated SNP, SORT1, to be elevated in AAA SMCs compared to control
Cheng et al. (21)	2022	Analysis of murine elastase- and CaCl_2_-induced AAA, and human AAA scRNA-seq databases, compared to mRNA microarray expression datasets	CaCl_2_ • Clusters: 15 Elastase • Clusters: 17	• Murine scRNA-seq datasets show the majority of infiltrating immune cells to be monocytes/macrophages • Functional enrichment, PPI analysis, and WGCNA of monocyte/macrophages pseudocells was performed on murine AAA scRNA-seq datasets to identify key functional pathways of Mo/Mø • DEG analysis of the two murine AAA datasets identified elevated expression of *Thbs1*, *Il1b*, and *Clec4e,* particularly within Mo/Mø. In the human AAA scRNA-seq database, these genes were also found to colocalize with CD68, a common macrophage marker
Ruan et al. (22)	2022	Re-analysis of human AAA scRNA-seq database, GSE166676	• Clusters: 11	• *PTPN22*, the gene encoding for protein tyrosine phosphatase non-receptor type 22, was identified as a potential AAA biomarker during cross-comparisons of GO public datasets. Analysis of the GSE166676 scRNA-seq database demonstrated upregulation of *PTPN22* within AAA tissues. Re-clustering using lineage-specific biomarkers identified the cells expressing higher *PTPN22* as immune cell origin. Subpopulation analysis defined these cells as T cells, NK cells, and B cells • Analysis of VSMCs *via ACTA2* expression showed AAA VSMCs to have higher expression of *PTPN22*

Ang II, angiotensin II; DC, dendritic cell; DEG, differentially expressed gene; EC, endothelial cell; Fib, fibroblast; GO, gene ontology; Maph or Mø, macrophage; Mo, monocytes; PPI, protein-protein interaction; SMC, smooth muscle; VSMC, vascular smooth muscle cell.

**Figure 1:**
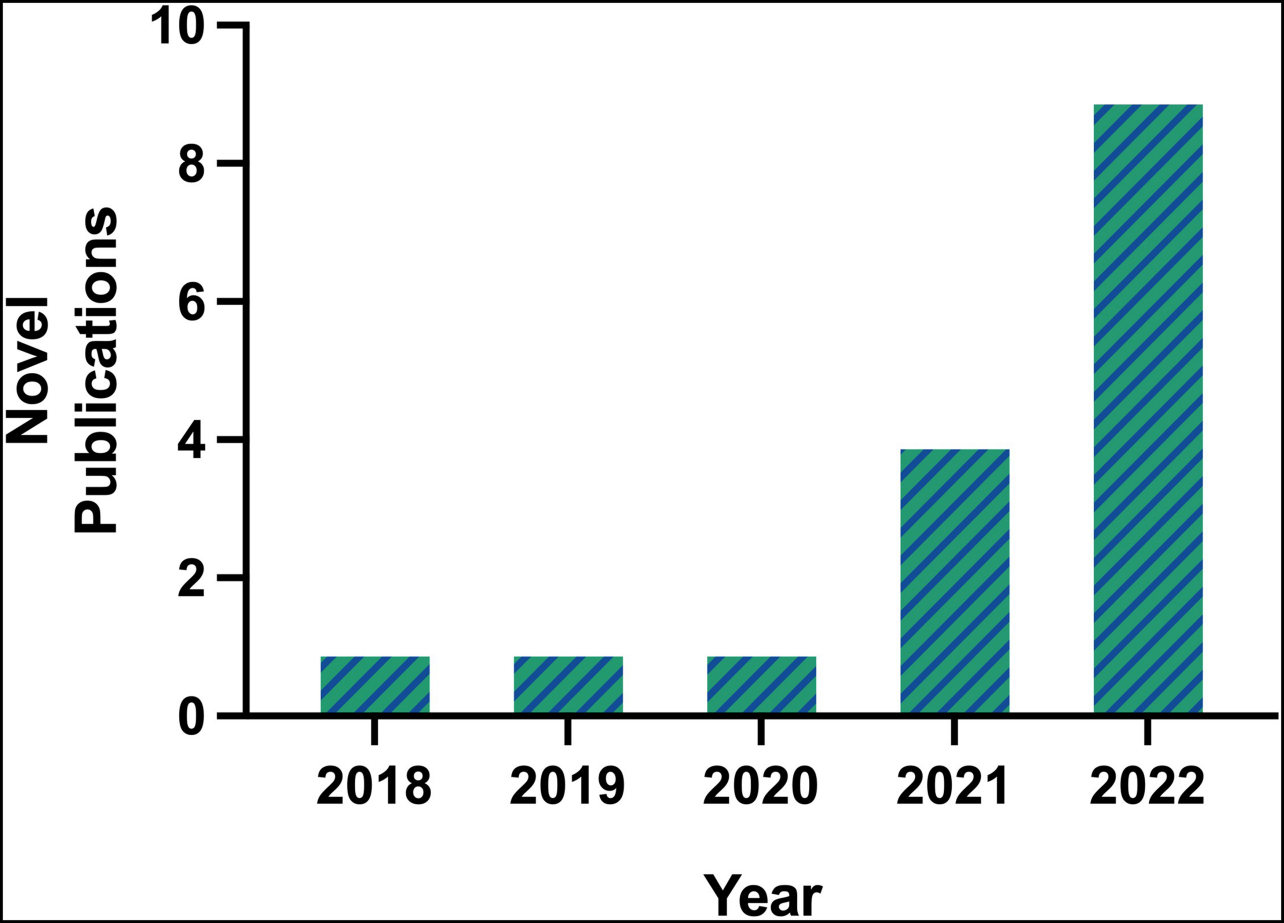
Number of novel publications on scRNA-seq in AAA available on PubMed prior to 2023, according to inclusion and exclusion criteria.

The first publication utilizing scRNA-seq technology for the study of AAA was by Hadi et al. in 2018, using an angiotensin II (Ang II)-induced AAA murine model (6). A single article per year on the topic of scRNA-seq in AAA was published between 2018 and 2020. By 2021, popularity of scRNA-seq in AAA began to rise as 4 manuscripts were published, one by Davis et al. which is the first and only study to use this technology on human AAA tissues (11). In 2022, 9 manuscripts were published (excluding the 2 articles previously mentioned), resulting in a near exponential increase in new publications per year (Figure 1). The greater number of publicly accessible datasets allowed for more analytical research, demonstrated by 5 of the 6 database studies being conducted that year.

The currently available AAA scRNA-seq databases were derived from analyzing human and experimental aneurysmal aortic tissues. Three models of murine aneurysm that have been analyzed by scRNA-seq include the Ang II osmotic pump model, adventitial elastase model, and perivascular calcium chloride (CaCl_2_) models. All of these mouse studies used young male mice. In the human dataset, 2 of the 4 AAA tissue samples were from female patients, while both controls (*n* = 2) were acquired from male patients undergoing aortobifemoral bypass (11).

Of the database analysis studies, most compared the human dataset to murine AAA sets, or compared scRNA-seq data to other available genetic data, such as mRNA microarray expression datasets. Most studies reclustered available datasets and utilized differentially expressed genes (DEGs) to identify a specific sequence of interest. One study utilized CellChat analysis to determine intercellular communication pathways by identifying outgoing and incoming signals between cell clusters (17).

Consistent with the wide use of the Ang II model by other approaches, 4 of the original scRNA-seq studies were conducted with this model (Table 1). Each of these studies used *ApoE^−/−^* mice that were infused with Ang II (1,000 ng/kg/min) for 28 days, and primarily focused their analysis on a particular tissue cell type (6, 12, 13, 15). The majority of the published murine AAA scRNA-seq studies were performed on the suprarenal aortic tissue segments when the aneurysmal dilation was well established. While the study by Yang and colleagues examined the transcriptomic profiles of infrarenal aortic tissues acutely following the aneurysm induction by CaCl_2_ (9), Zhao and colleagues examined both 7- and 14-day tissues following aneurysm induction by elastase (10). Despite of using different AAA models, pathological stages, and aortic segments, all of the investigative groups of the published scRNA-seq studies discovered multiple cellular types and several commonly altered signaling pathways including those responsible inflammation, vascular smooth muscle cell phenotype changes and aortic wall remodeling (Tables 1,2).

## Discussion

This brief systematic review serves as a resource for the current status of scRNA-seq in AAA. As identified above, scRNA-seq is gaining popularity and utility in AAA, as most studies have been conducted within the past year. Overall, this technology validates the existing understandings of the pathophysiologic mechanisms involved in AAA. Collectively, the scRNA-seq studies highlighted the importance of known pathological processes including inflammation, negative remodeling of extracellular matrix proteins, and phenotypic changes of vascular smooth muscle cells. However, with its capability of resolving transcriptome at a single cell resolution, scRNA-seq additionally provides a clear view on the heterogeneity of each of the major cell types found in aneurysmal tissues. Furthermore, the development of various bioinformatic analytical tools enables delineation of the specific interactions involved within complex pathways and predictions of cellular communications. For example, the scRNA-seq analysis conducted by Hadi et al. provides the precise mechanism infiltrated macrophages activate vascular smooth muscle cells to upregulate matrix metalloprotease-3 transcription and activation, which takes the known inflammatory response to a much deeper depth (6). Similarly, Davis et al.'s study recapitulates the critical involvement of inflammatory macrophages involved in AAA development, but also provides a molecular path through which macrophages transform (11).

Evaluating AAA using scRNA-seq has its limitations. In terms of scRNA-seq technology, evaluating changes in RNA at the transcriptome level does not necessarily reflect similar changes at the proteomic level. The high specificity of scRNA-seq comes at the cost of reduced sensitivity compared to bulk RNA sequencing, meaning low-abundance transcripts or rare cell phenotypes may be undetected or unrecognized using this method. Additionally, scRNA-seq typically generates low read coverage per cell and often uses short-read sequencing, which can make it difficult to identify alternative splicing events that occur at low levels. The manipulation of cells during ex vivo isolation may also lead to alterations in RNA prior to analysis. Using scRNA-seq for the evaluation of AAA is restricted by the limitations of current animal models and scRNA-seq databases. In mice, the available AAA models utilize only male mice in all the published scRNA-seq work. Although the majority of AAA occur in men, women, particularly postmenopausal women, are also affected by this highly lethal disease. The exclusion of female animals in the scRNA-seq study design diminishes the ability to evaluate sex differences in AAA. It is therefore a plausible progress made by Davis and colleagues to include AAA samples from female patients (11). However, it remains challenging to determine the optimal ratio between male and female samples, considering AAA disproportionally affects males.

In summary, scRNA-seq is a powerful research tool which enables unbiased investigation of AAA pathophysiology. Additional datasets from human AAA patients and controls are warranted. Further comparisons between human and murine datasets as well as between male and female datasets are useful in determining which of the complex molecular pathways are likely therapeutic targets.
